# The anterior cingulate cortex and pain processing

**DOI:** 10.3389/fnint.2014.00035

**Published:** 2014-05-05

**Authors:** Perry N. Fuchs, Yuan Bo Peng, Jessica A. Boyette-Davis, Megan L. Uhelski

**Affiliations:** ^1^Department of Psychology, University of Texas at Arlington, ArlingtonTX, USA; ^2^Department of Biology, University of Texas at Arlington, ArlingtonTX, USA; ^3^Department of Psychology, York College of Pennsylvania, YorkPA, USA; ^4^Department of Diagnostic and Biological Sciences, University of Minnesota, MinneapolisMN, USA

**Keywords:** anterior cingulate cortex, pain, pain affect, place escape, place avoidance

## Abstract

The neural network that contributes to the suffering which accompanies persistent pain states involves a number of brain regions. Of primary interest is the contribution of the cingulate cortex in processing the affective component of pain. The purpose of this review is to summarize recent data obtained using novel behavioral paradigms in animals based on measuring escape and/or avoidance of a noxious stimulus. These paradigms have successfully been used to study the nature of the neuroanatomical and neurochemical contributions of the anterior cingulate cortex (ACC) to higher order pain processing in rodents.

## Introduction

In 1968, Melzack and Casey (1968) proposed a model in which the neural structures that compose the brainstem reticular formation and limbic system are involved in the emotional and motivational determinant of pain, and that this system was separate from the neural structures that are involved in processing the sensory and discriminative dimension of pain. This proposal was based on the experience of pain, which consists of both affective and sensory components. Although the affective component of pain underlies the suffering that accompanies many persistent pain states and has been the subject of a large amount of human research (Shackman et al., 2011; Davis and Moayedi, 2013; Wager et al., 2013), this critical feature has historically not been the focus of pre-clinical basic research. Therefore, the purpose of the present paper is to summarize the literature on the separation of the discriminative and affective components of pain. The focus is on the role of the anterior cingulate cortex (ACC) in pain processing, with specific consideration of recent pre-clinical research using behavioral methodologies to explore the affective dimension of pain in rodents.

The neuromatrix theory of pain (Melzack, 2001, 2005) integrates the cognitive-evaluative, sensory-discriminative, and motivational-affective components proposed by Melzack and Casey (1968) and suggests that pain is a complex experience produced by a unique neurosignature of a widespread brain neural network. A number of different lines of evidence indicate that the ACC is a critical brain region that is part of the neuromatrix involved in pain processing (Vogt, 1985; Sikes and Vogt, 1992; Vogt and Sikes, 2009; Shackman et al., 2011; Iannetti et al., 2013; Wager et al., 2013). For instance, ACC neuronal activity increases during escape from a noxious thermal stimulus and in direct response and/or anticipation of noxious, but not non-noxious chemical, mechanical and thermal stimuli (Hutchinson et al., 1999; Koyama et al., 2001; Iwata et al., 2005). In humans, surgical cingulotomy and cingulectomy, or transection of the cingulum bundle and cingulate cortex, respectively, decreases the affective response to noxious stimuli, but does not alter the ability to localize the unpleasant stimulus. Cingulotomy has been performed with success (>50%) for the treatment of intractable cancer pain (Ballintine et al., 1967; Pereira et al., 2013), reflex sympathetic dystrophy (Santo et al., 1990), upper abdominal/lower thoracic pain (Sherman, 1973), low back pain (Sherman, 1973), and neuropathic pain (Boccard et al., 2014). In the study by Ballintine et al. (1967), 22 of 35 patients with pain from terminal cancer and 48 of the 77 patients with nonmalignant pain obtained significant relief from pain following cingulotomy. Brain imaging studies consistently report increased ACC neuronal activity preceding and during the presentation of an acute noxious stimulus or during persistent pain conditions (Cifre et al., 2012; Yuan et al., 2013). Hypnotic suggestions to selectively decrease pain affect prior to and during noxious stimulation resulted in decreased ratings of pain unpleasantness, but not pain intensity (Rainville et al., 1997). Manipulating pain unpleasantness by hypnotic suggestion also changed the regional cerebral blood flow (rCBF) in the ACC, but not in the somatosensory cortex, providing evidence that the ACC is involved in the processing of pain-related affect, but not in the sensory processing of noxious stimulation (Rainville et al., 1997). In addition to hypnotic suggestion, real-time fMRI studies have shown that both healthy controls and chronic pain patients can be trained to decrease activity in the ACC for relief of pain, with greater “control” of neurofeedback corresponding to lower pain ratings (Chapin et al., 2012), although as noted by Birbaumer et al. (2013), the use of real-time fMRI to regulate brain metabolism for pain control requires additional verification.

Exploring the role of the ACC in affective pain processing using animal models has only recently been performed. Historically, most animal studies that have examined supraspinal focal brain stimulation or microinjection of drugs into discrete nuclei have measured *reflexive* behavioral response to acute noxious stimulation. Indeed, activation of various subcortical, brainstem, and spinal cord systems produce antinociception as revealed by an increase in the threshold or latency to respond to noxious stimulation. In these studies, it is assumed that manipulations of limbic system structures alter pain processing through selective modulation of pain affect (Fuchs et al., 1996; Donahue et al., 2001). However, the majority of past and current behavioral paradigms used in animals cannot provide definitive information about the aversive and unpleasant qualities of a persistent pain condition. Thus, it has been difficult to distinguish the affective/motivational from the sensory/discriminative components of pain processing in animal models. In addition, any study that attempts to examine higher order processing of noxious input in animals must address the exact nature of pain affect. For instance, the affective (i.e., worrisome, cruel, fearful, terrifying, etc.) nature of chronic pain in humans is dissociable from the sensory (i.e., shooting, stabbing, pinching, cramping, etc.) nature of the condition by the descriptors that patients select on the McGill Pain Questionnaire (Melzack, 1975). In animal studies, the precise nature of pain affect is more difficult to define. We believe that affect, as it relates to noxious input, is certainly a negative hedonic state that can be identified in one way as aversion to a noxious stimulus.

## Behavioral paradigms in animals

The idea that a non-human animal’s natural avoidance of a hypersensitive area could be utilized to better understand pain is a relatively recent development in the literature. Few early references make mention of this finding. For example, Black (1990) made the informal observation that animals will perform an avoidance reaction to anything that touched the afflicted area following trigeminal nerve denervation. Vos et al. (1994) found “prolonged aversive behavior” in response to stimulation of the innervated facial area following chronic constrictive injury to the infraorbital nerve. It has only been more recently that investigators have begun to study the complexity of nociceptive processing by using behavioral methodologies focused on the concept that escape and/or avoidance of a noxious stimulus is a clear indication that animals find the stimulus aversive (Fuchs, 2000).

**Figure 1:**
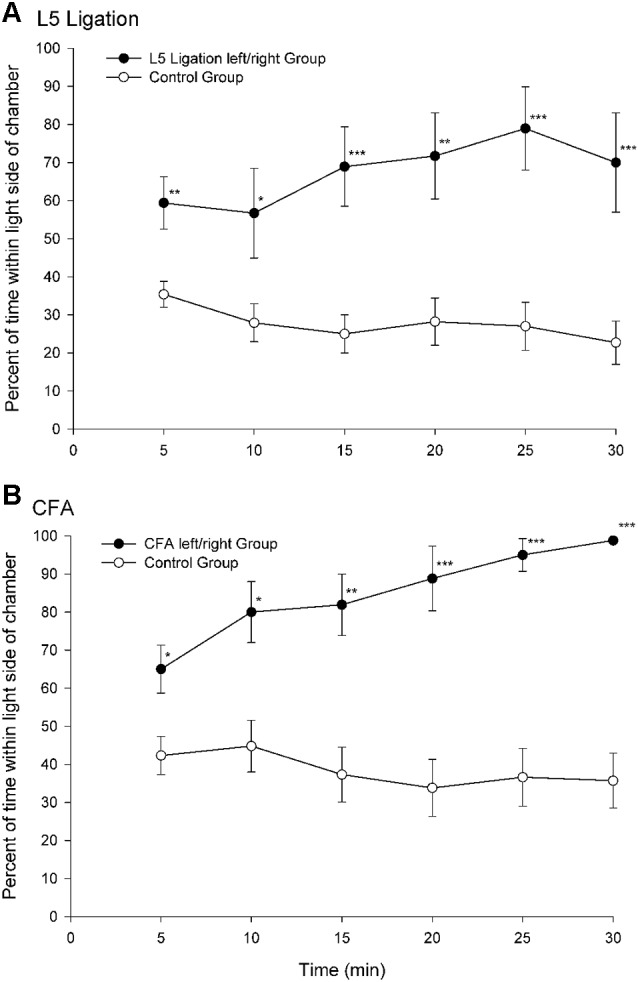
**Percentage of time (mean ± SEM) for each 5 min test interval spent in the light side of the test chamber for animals following peripheral nerve damage (L5 ligation, A) or inflammation (subcutaneous complete Freund’s adjuvant (CFA), B)**. When mechanical stimulation of the ligated or inflamed paw was associated with the preferred dark area of the chamber animals spend more time in the light area of the chamber indicating that the animals find stimulation of the hypersensitive paw aversive (Reprint from LaBuda and Fuchs, 2000a).

A paradigm that was developed in our laboratory utilizes escape/avoidance behavior by allowing animals to associate the application of a mechanical stimulus to an experimentally induced hyperalgesic paw with the preferred dark area of a test chamber (LaBuda and Fuchs, 2000a). In this procedure, animals are allowed to “choose” the location where the noxious stimulus is applied via their location in a test environment. The basic paradigm involves the use of a chamber that is equally divided into two distinct compartments. One side of the chamber is dark and the other side is light. Under normal conditions, the natural preference for animals is the dark area of the chamber. In experimental studies, hypersensitivity is generated in one hindpaw using procedures to produce either unilateral nerve injury or inflammation. During behavioral testing, noxious input is generated by applying a suprathreshold mechanical stimulus to the plantar surface of the hindpaws. The hypersensitive paw is mechanically stimulated when the animals are located in the dark side of the chamber and the normal (non-manipulated) paw is stimulated when the animals are located in the light side of the chamber. Escape/avoidance behavior is measured as a shift from the preferred dark area of the chamber to increased time spent within the non-preferred light area of the chamber. Control animals spend about 20–40% of the time in the light side of the chamber (Figure 1). However, experimental groups demonstrate escape/avoidance behavior toward the dark side of the chamber, and a shift in preference to the light side of the chamber, spending 60–100% of the time in the light side of the chamber.

This place escape/avoidance paradigm (PEAP) has been shown to be sensitive to pharmacological treatments such that manipulations which decrease mechanical hypersensitivity (i.e., gabapentin and morphine) also decrease escape/avoidance behavior (LaBuda and Fuchs, 2000b). Interestingly, doses of aspirin and morphine that do not attenuate mechanical hypersensitivity have also been shown to selectively attenuate escape/avoidance behavior (LaBuda and Fuchs, 2001; LaGraize et al., 2006), implying that the paradigm is sensitive to a unique aspect of pain processing. It should be noted that individual differences in baseline anxiety levels do not significantly modulate pain processing related to pain affect and motivation (Wilson et al., 2007). In summary, the occurrence of escape/avoidance behavior supports the notion that animals find stimulation of the hypersensitive paw aversive, and that when they have a “choice” they will perform purposeful behavior to minimize stimulation of the hypersensitive paw. This conclusion is further supported by the finding that conditioned place avoidance (F-CPA) can be induced to a compartment of an apparatus that is associated with a formalin injection (Johansen et al., 2001) and conditioned place preference can be used to reveal the presence of ongoing pain and pain relief (King et al., 2009; Navratilova et al., 2013).

## The ACC and pain processing in animals

**Figure 2:**
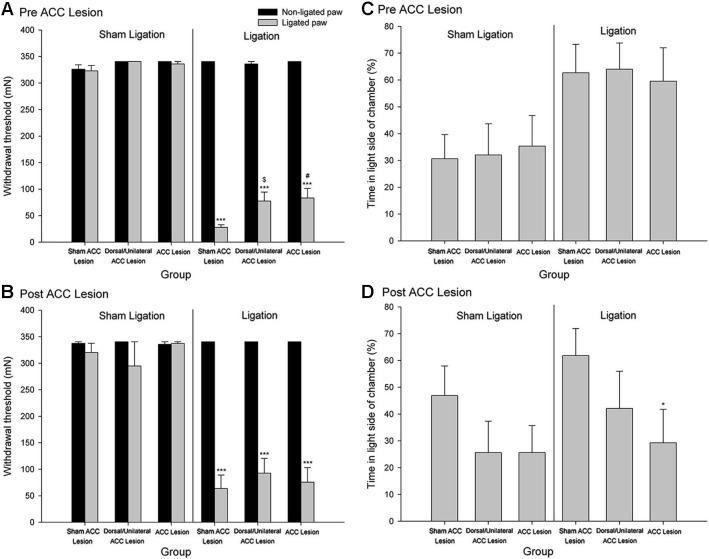
**Mechanical Paw Withdrawal Threshold. (A)** Mean ± SEM mechanical paw withdrawal threshold *prior* to stereotaxic surgery for sham ACC, dorsal/unilateral ACC lesion, or bilateral ACC lesion for animals with sham ligation or ligation of the L5 spinal nerve. **(B)** Mean ± SEM mechanical paw withdrawal threshold *following* stereotaxic surgery for sham ACC, dorsal/unilateral ACC lesion, or bilateral ACC lesion for animals with sham ligation or ligation of the L5 spinal nerve. *** *p* < 0.001 versus right paw control; ^#^ *p* < 0.001 versus sham ACC lesion ligated paw; *p* < 0.01 versus sham ACC lesion ligated paw. *Place Escape/Avoidance Behavior*. **(C)** Mean ± SEM percentage of time within the light side of the test chamber *prior* to stereotaxic surgery for sham ACC, dorsal/unilateral ACC lesion, or bilateral ACC lesion for animals with sham ligation or ligation of the L5 spinal nerve. **(D)** Mean ± SEM percentage of time within the light side of the test chamber *following* stereotaxic surgery for sham ACC, dorsal/unilateral ACC lesion, or bilateral ACC lesion for animals with sham ligation or ligation of the L5 spinal nerve. * *p* < 0.05 versus sham ACC lesion ligation control (Reprint from LaGraize et al., 2004b).

Although as is discussed above, evidence suggests that the ACC processes the affective/motivational component of pain, there has, until recently, been a paucity of basic research exploring the neural mechanisms underlying affective/motivational nociceptive processing (Borszcz and Streltov, 2000). Behavioral paradigms, such as the place escape/avoidance test, have permitted the separate assessment of affective and sensory components of the pain experience and have led to the examination of the underlying neuroanatomical and neurochemical processes that might be involved in modulating pain affect. We have found that electrolytic lesion of the ACC differentially alters mechanical hypersensitivity and escape/avoidance behavior (LaGraize et al., 2004b). As seen in Figure 2, animals that received a nerve injury induced by tight ligation of the L5 spinal nerve spent 60–65% of the time in the light area of the test chamber prior to lesion of the ACC. Following the lesion of the ACC, escape/avoidance behavior in L5 ligated animals decreased to approximately 30%, similar to the control group. Of additional interest is the lack of effect of ACC lesion on mechanical hypersensitivity (Figures 2A and 2B). A similar effect has been reported with the use of kainic acid lesions of the ACC, which reduced pain behaviors associated with subcutaneous injection of bee venom (Ren et al., 2008) without altering hypersensitivity to mechanical stimuli. Another study conducted in our laboratory demonstrated that escape/avoidance behavior was associated with the expression of cFos, a marker of neuronal activation, in the ACC (Uhelski et al., 2012b). Following unilateral intraplantar injection of carrageenan, subjects with the highest percentage of time spent in the light side of the chamber tended to demonstrate the highest level of cFos expression. Importantly, cFos expression was unrelated to the degree of hypersensitivity in the paw as measured by mechanical threshold testing. Conversely, a recent study conducted in our laboratory found that bilateral lesions to the area of the somatosensory cortex involved in processing sensory information from the hind paws interfered with the expression of mechanical hypersensitivity in assessments of mechanical withdrawal thresholds but did not inhibit escape/avoidance behavior in the PEAP (Uhelski et al., 2012a). This suggests that the processing of pain affect in the limbic system, including the ACC, is functionally distinct from the processing of sensory information, and that noxious stimuli can still be perceived as unpleasant in the absence of information regarding the location and intensity of the stimulus. Our findings are also supported by using other novel paradigms, such as formalin-induced conditioned place avoidance (Johansen et al., 2001) and conditioned place preference (Qu et al., 2011).

**Figure 3:**
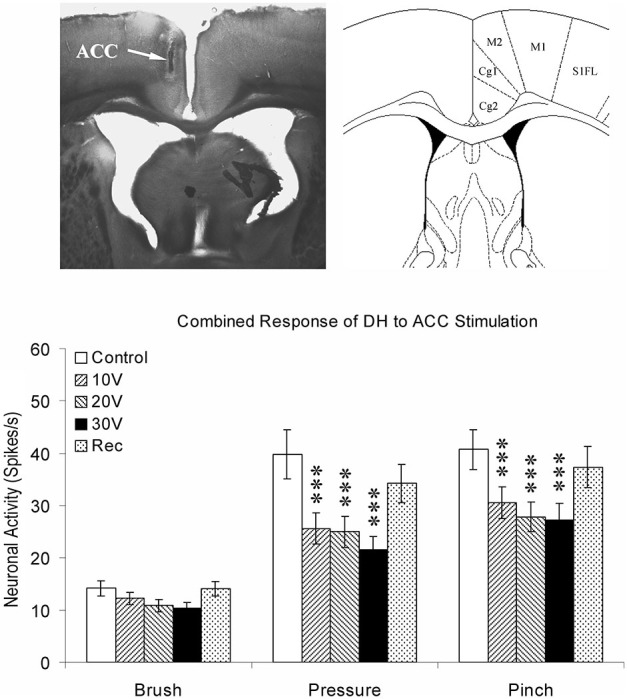
**Summary of ACC modulation of spinal dorsal horn neuronal responses**. The site of stimulating electrode was verified by brain histology. The arrow points to the anterior cingulate cortex (Cg1) on the left according to the coordinates provided by Paxinos and Watson (1998) on the right. Responses of spinal dorsal horn (DH) neurons to mechanical stimuli (Brush, Pressure, and Pinch) during ACC stimulation. The results are combined for ipsilateral and contralateral ACC stimulation. *** *p* < 0.001 (Modified from Senapati et al., 2005; with permission).

**Figure 4:**
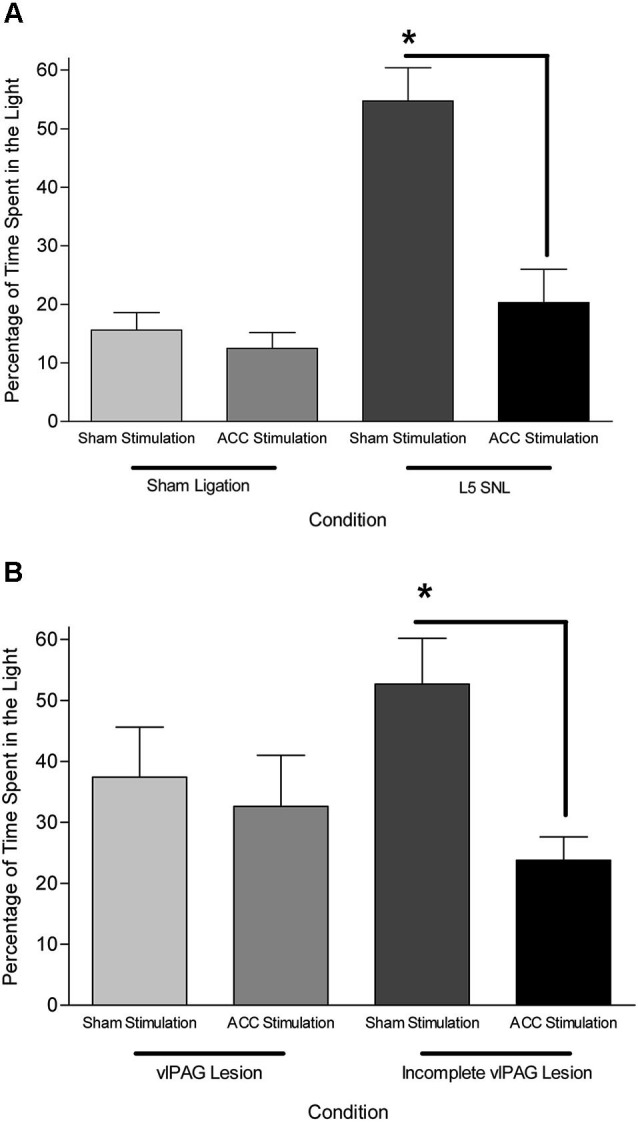
**(A)** Mean (± SEM) time spent in the light side of the PEAP chamber in sham vlPAG lesioned animals. L5 SNL, Sham ACC stimulated animals avoided noxious hindpaw stimulation significantly more than sham ligated, sham ACC stimulated animals. ACC stimulation did not have an effect in sham ligated animals, but significantly attenuated the avoidance behavioral response in L5 SNL animals. * *p* < 0.05 compared to sham ligated + sham ACC stimulation treated animals. **(B)** Mean (± SEM) time spent in the light side of the PEAP chamber in L5 SNL animals with vlPAG lesions or incomplete vlPAG lesions. L5 SNL, Sham ACC stimulated animals with an incomplete lesion of the vlPAG avoided noxious hindpaw stimulation significantly more than L5 SNL, ACC stimulated animals with an incomplete vlPAG lesion. There was not a significant avoidance response following vlPAG lesions in ACC stimulated, L5 SNL animals. * *p* < 0.05 compared to sham ligated + sham ACC stimulation treated animals (Reprint from LaBuda and Fuchs, 2005).

Data from neuroanatomical and behavioral pharmacological studies strongly suggest the existence of a supraspinal pain suppression system at the level of the periaqueductal gray (PAG). Research investigating this system indicates that activation of the ACC might recruit the pain suppression system at the level of the PAG. Indeed, focal brain stimulation of the ACC has been shown to inhibit the response of dorsal horn neurons to mechanical stimulation (Figure 3; Senapati et al., 2005; Ma et al., 2011), although evidence for facilitation of a nociceptive behavioral response has also been reported (Calejesan et al., 2000). We conducted an experiment to determine whether the effect of ACC activation was due to the activation of an endogenous pain suppression system at the level of the PAG (LaBuda and Fuchs, 2005). As seen in Figure 4, focal electrical stimulation of the ACC significantly reduced escape/avoidance behavior as revealed by animals spending less time in the light side of the chamber. Of additional interest is the finding that lesions of the vlPAG significantly attenuated the effect of ACC activation. Animals with lesions of the vlPAG and stimulation of the ACC spent more time in the light side of the chamber compared to animals that had no lesions (or incomplete lesions) of the vlPAG and received stimulation of the ACC. In no instance was there a change in the mechanical paw withdrawal threshold. The results of this study further support the notion that sensory and affective pain processing can be differentiated using the PEAP.

**Figure 5:**
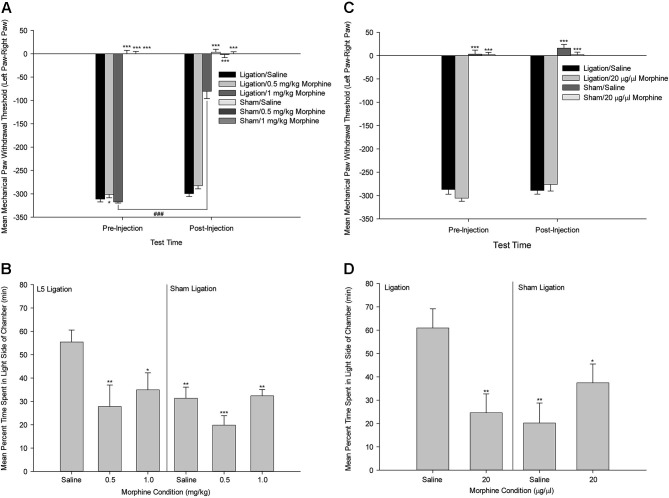
**Systemic Morphine. (A)** Mean ± SEM mechanical paw withdrawal threshold difference scores (left paw–right paw) prior to and following drug administration (Saline, 0.5 mg/kg morphine, 1.0 mg/kg morphine) for animals with sham ligation or ligation of the L5 spinal nerve. **(B)** Mean ± SEM percentage of time within the light side of the test chamber for the duration of the 30-min test period following drug administration (Saline, 0.5 mg/kg morphine, 1.0 mg/kg morphine) for animals with sham ligation or ligation of the L5 spinal nerve. * *p* < 0.05 versus Ligation/saline at that time point; ** *p* < 0.01 versus Ligation/saline at that time point; *** *p* < 0.001 versus Ligation/saline at that time point; ^###^ *p* < 0.001 versus same group at pre-injection time point. **(C)** Mean ± SEM mechanical paw withdrawal threshold difference scores (left paw–right paw) prior to and following microinjection (Saline, 20 µg/µl morphine) into the ACC for animals with sham ligation or ligation of the L5 spinal nerve. **(D)** Mean ± SEM percentage of time within the light side of the test chamber for the duration of the 30-min test period following microinjection (Saline, 20 µg/µl morphine) into the ACC for animals with sham ligation or ligation of the L5 spinal nerve. * *p* < 0.05 versus Ligation/saline; ** *p* < 0.01 versus Ligation/saline; *** *p* < 0.001 versus Ligation/saline (Reprint from LaGraize et al., 2006).

This work suggests that one possible mechanism of action by which the ACC selectively modulates pain affect is via the mu-opioid receptor system. This idea is based on the evidence that the ACC has a high density of opioid receptors (Lewis et al., 1983; Mansour et al., 1987; Kujirai et al., 1991; Vogt et al., 1995), and that activation of the ACC mu-opioid receptor system during sustained pain is negatively correlated with McGill Pain Questionnaire affective scores (Zubieta et al., 2001) and the Positive and Negative Affectivity Scale (Zubieta et al., 2003). In a more recent study, we tested the functional role of the ACC opiate system in the selective modulation of pain affect in an animal model of neuropathic pain utilizing the methods of mechanical paw withdrawal threshold and escape/avoidance behavior testing (LaGraize et al., 2006). Systemic administration of low dose morphine produced a selective attenuation of pain affect, as indicated by a decrease in the amount of time that animals spent in the light side of the chamber in nerve damaged animals, with no alteration of mechanical paw withdrawal threshold (Figure 5). Supraspinally, morphine microinjection into the ACC again produced a selective decrease in escape/avoidance to mechanical stimulation of the hyperalgesic paw with no change of mechanical paw withdrawal threshold. Since the ACC has been implicated in learning and memory processes (Peretz, 1960; Kimble and Gostnell, 1968; Seamans et al., 1995; Engström et al., 2013), it is possible that morphine administration interferes with the acquisition and retention of information in the PEAP rather than decreasing the negative hedonic value of the mechanical stimulus. Impaired performance following morphine administration has been reported in tests of spatial memory in the Morris water maze (McNamara and Skelton, 1992) and the radial arm maze (Spain and Newsom, 1991). However, it should be noted that the effect of morphine on the radial arm maze requires chronic high dose administration (up to 40 mg/kg) that is most likely associated with sedation and impairment in task performance rather than with interference of working memory (Spain and Newsom, 1991). Other investigators report biphasic results in rats such that lower doses of morphine enhance while higher doses impair memory (Ageel et al., 1976; Galizio et al., 1994). Avoidance responding has been reported to be unaltered following morphine administration at doses that inhibit reflexive withdrawal responding in non-human primates (Yeomans et al., 1995). Further, impairment of learning and memory function is typically associated with manipulations to the more posterior regions of the cingulate cortex. It is therefore unlikely that our results can be explained as a failure to learn, however ongoing research is comparing the role of the anterior versus the posterior cingulate cortex on spatial learning and pain processing.

## Additional considerations

Clinically, early research indicated that cingulotomies or cingulectomies have a minimal impact on intelligence (Foltz and White, 1962; Sherman, 1973). In addition, electrical stimulation of the ACC in humans was also shown to have no effect on emotional expression, speaking ability or comprehension (Talairach et al., 1973). More recent work has implicated the dACC in “cognitive control” (Paus, 2001; Sheth et al., 2012), however such processes were found to be not impaired in human subjects with damage to the dACC (Fellows and Farah, 2005). Although ACC lesions in rats can interfere with certain rodent tests of learning and memory, the damage is usually more posterior, extensive, and includes damage to the cingulum bundle and other surrounding tissue including the hippocampus, a region implicated in learning and memory processes. Taken together, the most parsimonious interpretation of the present results is that the ACC is involved in processing the affective component of pain, and likely a component of a system that is responsible for expressing affect and engaging goal-directed behavior (Paus, 2001; Vogt and Sikes, 2009; Shackman et al., 2011; Misra and Coombes, 2014).

Given the reduction in aversion behavior produced by manipulations of the ACC, we have proposed that the effect of ACC lesions on tests of learning and memory most likely reflect a reduced affective response to noxious footshock, a stimulus commonly used to examine learning and memory in rodents. Bilateral cingulate cortex aspiration in rats significantly impairs the acquisition of a two-way conditioned avoidance behavioral response to noxious footshock (Peretz, 1960; Kimble and Gostnell, 1968). Rats with bilateral ACC lesions also failed to demonstrate conditioning in a paradigm where a tone was paired with the application of a noxious laser stimulus (Kung et al., 2003). However, ACC lesioned animals are not impaired in the acquisition of a visual discrimination task and the bar-pressing rate for animals under food deprivation over six 30-min sessions is higher for cingulectomized animals compared with control animals. From these studies, it is suggested that ACC damage impairs acquisition of conditioned avoidance but does not produce a general decrease of all drives (Peretz, 1960). However, excitotoxic lesion of the ACC was recently reported to decrease formalin conditioned place avoidance without altering electric foot-shock-induced place avoidance (Gao et al., 2004). Based on previous studies, the reason for the lack of alteration in electric foot-shock-induced place avoidance following ACC lesions reported by Gao et al. (2004) is unknown. Assuming that ACC lesions do alter conditioned avoidance to noxious footshock, we suggest that shock avoidance behavior is impaired not because of changes in memory mechanisms, but because the aversive nature of the shock is decreased following manipulation of the ACC. Future studies that examine the role of the ACC in learning and memory that utilize noxious stimulation must control for the important role of the ACC in the affective response to threatening and noxious information.

Activation of the ACC by electrical stimulation has been shown to reduce the aversive quality of tactile stimulation to the allodynic hindpaw in the PEAP. It is speculated that ACC stimulation produces its effects by inhibiting normal activity within a system that we (LaBuda and Fuchs, 2005) termed the negative affective pain processing system (NAPPS) to produce an effect that is similar to the effect seen after cingulectomy. Alterations in the NAPPS might explain why both electrical stimulation (LaBuda and Fuchs, 2005) and selective lesions of the ACC (LaGraize et al., 2004b) reduce the behavioral response to noxious stimulation of an allodynic hindpaw. However, a recent report has demonstrated that direct stimulation of the ACC in mice produces fear-like freezing responses and induces long-term fear memory (Tang et al., 2005). The authors speculated that excitatory activity in the ACC contributes to pain-related fear memory as well as descending facilitatory modulation of spinal nociception. Why stimulation in one instance decreases pain affect and in another instance contributes to pain-related fear memory remains to be determined.

An understanding of pain in the context of higher order cognitive processing has been suggested to provide a framework for the comparative assessment of anatomy, physiology and behavior (Allen, 2004; Allen et al., 2005; Gatchel et al., 2006). The recent work on the sensory and emotional aspects of pain experiences in rats provides a context in which the functional roles of different components of the phenomenology of pain can be investigated with respect to anatomy (particularly the role of the ACC), physiology (the effect of opioid substances), and behavior (avoidance of aversive contexts) (LaBuda and Fuchs, 2000a,b, 2005; LaGraize et al., 2004b, 2006). The approach is directed using an empirical research program based on a functional understanding of pain to allow comparisons to be drawn between the pain experiences of humans and those of other animals. The move from simple behavioral measures (stimulus-response) to more sophisticated behavioral techniques, along with advances in rodent fMRI methodology (Borsook et al., 2006) provides such an approach for investigating the roles that different dimensions of painful experiences might play in higher order processing. In addition to the avoidance techniques outlined above, it is worth noting that operant techniques provide a unique way to investigate the interplay between pain and other processes, including learning, memory, and attention. In one recent study, our lab demonstrated that a dose of morphine that was effectively analgesic but not strongly sedating was able to abate cognitive deficits resulting from an acute pain stimulus (Boyette-Davis et al., 2008). This approach provides a framework for answering a host of other relevant questions. For example, is long-term conditioning differentially affected by blocking the sensory and affective components of pain processing? Does treatment with morphine affect the ability of rats to learn about noxious stimuli? Would treated rats fail to learn associations between contextual cues and noxious stimuli, or is sensory awareness sufficient? Would the effect vary for different types of pain conditions (i.e., is sensory awareness sufficient for acute conditions but not chronic conditions?). Additionally, if given the choice, would rats learn to self-administer sensory and affective pain relief differentially?

Related to these ideas of learning are questions currently unanswered regarding the role pain may have in motivational drives. Humans suffering from chronic pain experience a multitude of personal issues (Gatchel et al., 2007) including depression, anhedonia, changes in appetite, etc., yet the underlying neuroanatomical and neurophysiological reasons for these experiences are not clearly understood. By utilizing a functional approach, these and other issues can be addressed. For example, do animals experiencing food deprivation and pain simultaneously choose to eat, or does the pain drive supersede the hunger drive? Research from our laboratory indicates that pain can modulate the hunger drive (LaGraize et al., 2004a). Is this choice differentially affected by blocking sensory and affective components of pain processing? Furthermore, is the loss of pain affect associated with loss of affect in other behaviors (i.e., mating, predator/prey, and maternal behaviors)? Do losses of pain affect versus sensory pain experience differentially modify these behaviors? The ability to investigate such questions at a functional level of analysis opens the door to much more detailed analyses of the importance of the ACC in these different aspects of painful experiences.

Future research will no doubt address these questions and also add to our understanding of the cellular changes occurring in the ACC and how these changes impact the pain neuromatrix. Previous research had indicated that painful stimuli can lead to increases in NMDA receptors in the ACC and that these increases are related to the formation of formalin-induced conditioned place avoidance (Lei et al., 2004; Li et al., 2009). Similar results have been found for the NMDAR ligand glutamate (Johansen and Fields, 2004). Other studies have supported this idea that increased glutamate signaling in the ACC is an important aspect of the persistence of many pain states (Wu et al., 2008; Yan et al., 2012; Zhou et al., 2014). LaGraize and Fuchs (2007) identified an important role of the ACC GABA system in modulating the affective component of pain. There is also evidence that altered expression of the neuropeptide cholecystokinin (CCK) in the ACC is associated with the induction of arthritis in rodents (Erel et al., 2004). CCK, which is expressed prominently by interneurons and some projection neurons of the ACC, is known to induce anxiety when administered experimentally to both humans and rodents (see Harro et al., 1993 for review). Administration is also associated with increased blood flow to the ACC in humans (Benkelfat et al., 1995), and there is some evidence that CCK is involved in the anti-nociceptive effect of opioids (see Ossipov et al., 2003 for review). The specific role of CCK in the expression of pain affect has not been fully examined; however, its prominence in the ACC suggests that it may play a significant role in pain processing. Metabolic changes, including alterations in protein kinases such as PKMζ (Li et al., 2010) and SIP30 (Han et al., 2014) have also been implicated to contribute to pain processing in the ACC. Understanding these cellular changes will guide researchers as they continue to investigate pain using a functional approach.

The goal of translational research is ultimately to use what is learned from basic research to improve the health and wellbeing of society. One recent advancement is that of optogenetics, a technique which allows for the expression of light-sensitive proteins in selected neurons through either breeding genetically modified rodents or the use of a viral vector in developed subjects. Early work in this field has demonstrated that this experimental technique can be used to activate or deactivate affected specific neurons simply by exposing the tissue to a specific frequency of blue light. Researchers examining the memory formation in the hippocampus reported that optogenetic inactivation of the ACC impaired fear memory recall in mice (Goshen et al., 2011). This suggests that optogenetic control of the ACC is possible, and there is great potential for novel experimental techniques in areas such as optogenetics and biomedical engineering to shed light on the role of specific neuronal populations in the ACC in pain processing. The creation of implantable devices could be the next step in long-term treatment for the vast array of chronic pain conditions which are difficult to treat with standard pharmaceutical regimens.

## Conclusion

The most parsimonious explanation of the behavioral studies in rodents is that the ACC is involved in the modulation and processing of pain affect, and that manipulations of the ACC alter the affective component of pain processing. Of primary interest is that recent behavioral paradigms in animals have measured escape and/or avoidance of a noxious stimulus, assuming that escape/avoidance behavior is evidence that animals find the noxious stimulus aversive. These paradigms have successfully been used to study the nature of the neuroanatomical and neurochemical mechanisms that underlie the processing of higher order pain processing in rodents and have strongly implicated the ACC as a critical brain structure.

One very important outcome of the recent findings is the indication that it is inappropriate to examine the role of the ACC in nociceptive processing in animals by relying on measures of reflexive behavior as a means to examine the aversive nature of persistent pain conditions. Experiments using only quantified mechanical thresholds or acute formalin injection behaviors can lead to the erroneous conclusion that the ACC does not affect supraspinal pain processing. Clearly, functional alterations of the ACC by lesion and neurochemical methods reduce the avoidance of noxious mechanical hindpaw stimulation as measured using the PEAP and formalin conditioned place avoidance paradigm. Sensory mechanisms of pain processing are clearly important but fail to highlight the mechanisms underlying the affect that accompanies many persistent pain conditions. Clinically, the sensation of pain can be treated by reducing the sensory input as well as by manipulating affective-motivational and cognitive factors (Melzack and Casey, 1968). Therefore, an understanding of the neural substrates mediating the affective processing of nociceptive stimulation should advance our knowledge of pain processing and contribute to advancements in therapeutic interventions to reduce the affective component of pain that accounts for the suffering so frequently seen in clinical conditions.

## Author contributions

All authors conceptualized the manuscript and were involved in the writing and editing the document.

## Conflict of interest statement

The authors declare that the research was conducted in the absence of any commercial or financial relationships that could be construed as a potential conflict of interest.
